# Anticoagulation Monitoring in Recurrent Venous Thromboembolism Associated With Antiphospholipid Antibodies and Subsequent Heparin-Induced Thrombocytopenia

**DOI:** 10.7759/cureus.118258

**Published:** 2026-10-20

**Authors:** George Bechir

**Affiliations:** 1 Internal Medicine, Franciscan Health, Munster, USA

**Keywords:** activated partial thromboplastin time, anticoagulation monitoring, antiphospholipid antibodies, heparin-induced thrombocytopenia, venous thromboembolism

## Abstract

A woman in her 20s with class III obesity and strongly positive antiphospholipid antibodies developed phlegmasia cerulea dolens and a new pulmonary embolus after recent catheter-directed thrombolysis. Before the first documented heparin administration, activated partial thromboplastin time was 95.5 s and international normalized ratio was 1.5. Eight subsequent paired samples showed activated partial thromboplastin times of 176 s to over 200 s with heparin anti-Xa activity of 0.20-0.66 IU/mL. Lupus anticoagulant testing was uninterpretable during anticoagulation, and international normalized ratio reliability was not independently established. Later thrombocytopenia prompted repeat heparin-induced thrombocytopenia testing despite an earlier negative result; a strongly positive immunoassay supported the treating team's diagnosis and transition to fondaparinux. This case illustrates the importance of selecting appropriate anticoagulation monitoring from the outset and reassessing heparin-induced thrombocytopenia when the clinical picture changes. Whether assay interference contributed to recurrent thrombosis remains unresolved.

## Introduction

Antiphospholipid syndrome (APS) is an acquired thrombotic disorder associated with persistent antiphospholipid antibodies. Anticoagulation is central to secondary prevention, but laboratory interpretation can complicate assessment of apparent recurrence [1,2]. Lupus anticoagulant (LA) can prolong phospholipid-dependent clotting assays in vitro because the antibodies bind phospholipid-bound proteins in the test system and delay clot formation, despite a thrombotic clinical phenotype [1]. Consequently, activated partial thromboplastin time (aPTT) may overestimate unfractionated heparin (UFH) effect when the baseline aPTT is prolonged. An appropriately calibrated anti-Xa assay provides an alternative for UFH monitoring [1]. International normalized ratio (INR) reliability must be assessed separately because the response to LA varies among thromboplastin reagents [2].

Heparin-induced thrombocytopenia (HIT) adds a distinct prothrombotic complication. Its assessment integrates platelet decline, heparin exposure, thrombosis, alternative explanations, and laboratory testing [3]. Antiphospholipid antibodies further complicate this assessment because positive HIT antigen tests without platelet activation have been reported in APS and systemic lupus erythematosus [4]. Persistent antibody positivity at least 12 weeks apart is relevant to APS classification, which must be distinguished from a treating team’s clinical diagnosis [5]. We report recurrent venous thromboembolism (VTE), marked aPTT/anti-Xa discordance, and a subsequent clinical diagnosis of HIT. This case illustrates the need to reassess anticoagulant exposure, select monitoring appropriate to each drug, and coordinate changing treatment requirements without assigning an unverified mechanism to recurrent thrombosis.

## Case presentation

A woman in her 20s, weighing approximately 113 kg with a body mass index of 44 kg/m², presented with pulmonary embolism (PE) after recent travel. She was a never-smoker with a documented family history of lupus and thrombosis. Travel duration and hormonal exposure were not established. Day 0 denotes her subsequent readmission. Table 1 summarizes the clinical timeline.

**Table 1:** Clinical timeline of thrombosis, anticoagulation, and platelet changes. Day 0 denotes readmission with phlegmasia cerulea dolens. Negative days refer to the initial admission. The timeline was compiled by the author from the supplied clinical records. DVT: deep venous thrombosis; PE: pulmonary embolism; UFH: unfractionated heparin; IVC: inferior vena cava; HIT: heparin-induced thrombocytopenia; aPTT: activated partial thromboplastin time; PT: prothrombin time; INR: international normalized ratio

Day	Clinical event	Treatment or monitoring
-13	Initial pulmonary embolism; baseline aPTT: 95.5 s and INR: 1.5	UFH initiated
-12	Catheter-directed thrombolysis	Subsequent warfarin with enoxaparin bridging
0	Readmission with phlegmasia and extensive DVT; earlier PE improved	UFH restarted
2	Mechanical thrombectomy	Procedural heparin; enoxaparin 1 mg/kg every 12 h
3	New pulmonary emboli; initial HIT immunoassay negative	Four enoxaparin administrations documented across days 2-3
4	IVC filter placed	Brief argatroban infusion, then UFH with anti-Xa monitoring
5-6	Marked aPTT/anti-Xa discordance	UFH titration and supplementary boluses
7	Transfer; platelets 300×10⁹/L	Continued specialist assessment
8	Rheumatology reassessment	Methylprednisolone stopped
9	aPTT: 176 s with anti-Xa: 0.34 IU/mL	Enoxaparin 80 mg every 12 h plus warfarin 2.5 mg daily
10	Platelets 84×10⁹/L	Reassessment of falling platelet count
11	Platelets 65×10⁹/L; repeat HIT sample collected	Enoxaparin and warfarin stopped; fondaparinux 10 mg daily
12	Positive HIT result reported, optical density: 2.562; platelets: 79×10⁹/L	Fondaparinux continued
13	Platelets: 115×10⁹/L; PT: 14.2 s, INR: 1.1	Early platelet recovery
Discharge plan	Hematology follow-up	Fondaparinux for three months, then reassessment for warfarin

At the first presentation, day -13, activated partial thromboplastin time (aPTT) was 95.5 s (reference: 25-36 s), PT 17.5 s, and international normalized ratio (INR): 1.5, approximately 3 h before the first documented unfractionated heparin (UFH) bolus. No anticoagulant, including pre-hospital low-molecular-weight heparin or a heparin flush, was documented before that sample, and no mixing study or factor assays were performed on it. The concurrent PT prolongation (INR: 1.5; antiphospholipid-panel PT: 20.0 s) was not explained by the available tests, because lupus anticoagulant (LA) alone rarely prolongs the PT to this degree and factor assays were not performed [2]. The referring laboratory's printed UFH aPTT range was 65-90 s. She received an 80 units/kg bolus based on adjusted weight, followed by UFH and catheter-directed thrombolysis on day -12. An infusion order displayed 9 units/kg/h on adjusted weight; a complete rate history was unavailable. INR was 1.8 on day -11 before the first listed warfarin order, and 2.1 on day -10. A 5 mg warfarin dose was ordered on day -11; administration was not independently established from the available extract. She was discharged on warfarin 2.5 mg daily with enoxaparin bridging. According to her history, injections ended on day -7; a clinic INR was approximately 2.0 three days later, prompting one additional 1.25 mg warfarin dose. The original outpatient INR and administration records were unavailable.

On day 0, right-leg pain, swelling, weakness, cyanosis, and coolness indicated phlegmasia cerulea dolens. Ultrasonography showed extensive occlusive proximal and distal deep venous thrombosis (DVT); chest CT showed improvement of the earlier PE. Notes differed regarding anticoagulation adequacy at readmission, and the original admission INR was unavailable for adjudication. UFH was restarted. An order displayed 3 units/kg/h on adjusted weight; serial aPTTs exceeded 200 s and subsequently decreased to 112.8 s. These entries did not establish the duration or reasons for individual infusion rates. Representative duplex ultrasound images from day 0 are shown in Figure 1A-1E. No clinical photograph of the limb was obtained during the admission.

**Figure 1:**
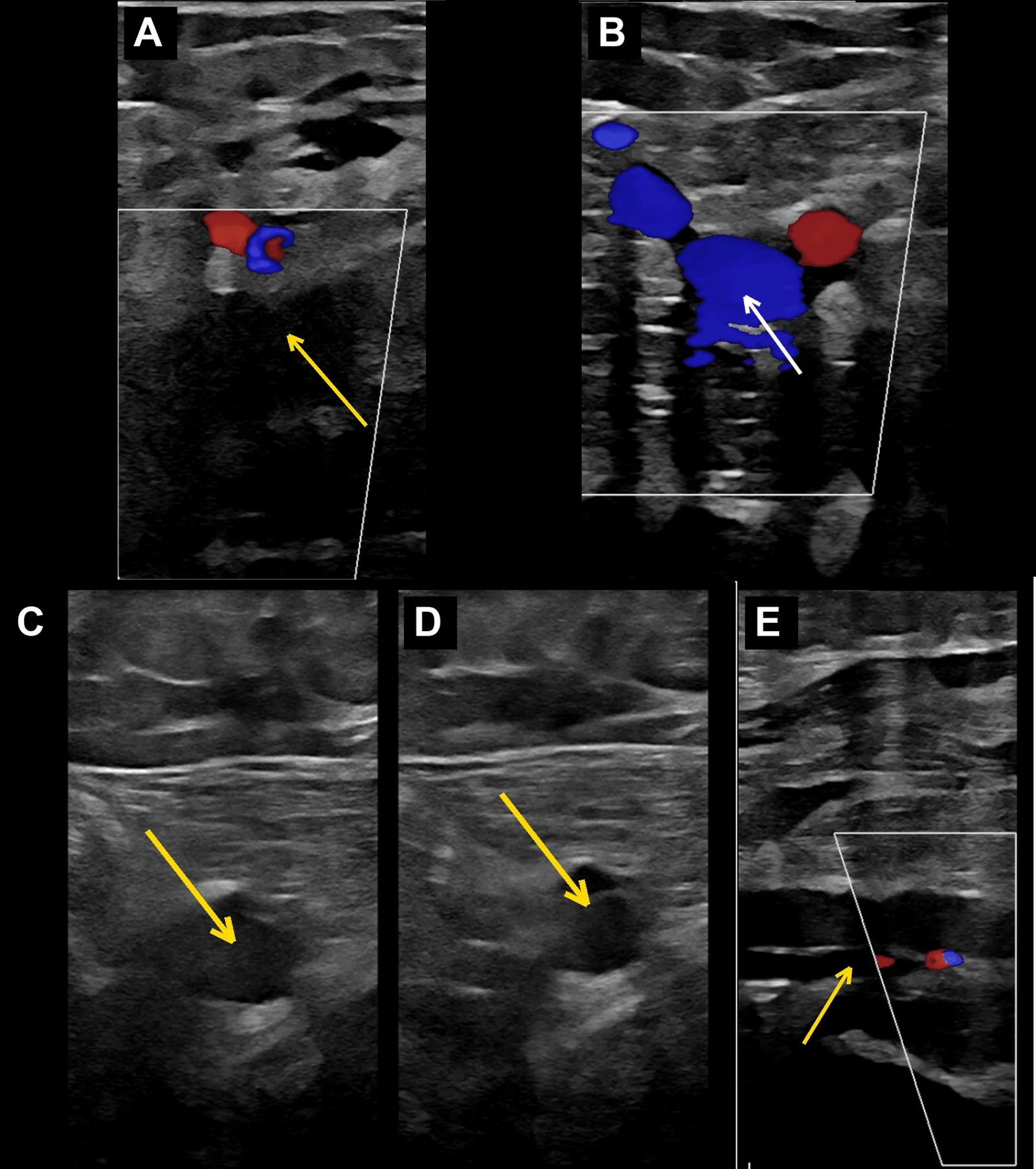
Duplex ultrasound of the lower extremities on the day of readmission (day 0). (A) Right common femoral vein, transverse view with color Doppler. The femoral artery shows color flow (red, flow toward the probe); the adjacent, distended common femoral vein (yellow arrow) shows no detectable color signal. (B) Left common femoral vein at the saphenofemoral junction, same examination, for comparison: the vein fills completely with color (white arrow), consistent with normal flow. (C, D) Right femoral vein at mid-thigh, transverse grayscale, without (C) and with (D) probe compression. The vein (yellow arrows) remains distended and non-compressible. (E) Right popliteal vein, longitudinal view with color Doppler. The popliteal artery shows color; the adjacent popliteal vein (yellow arrow) shows no detectable flow. Yellow arrows mark thrombosed segments; the white arrow in B marks the normal contralateral vein. The absence of color signal with a patent adjacent artery (A, E), along with non-compressibility (C, D), is consistent with occlusive deep vein thrombosis of the right lower extremity. Color indicates flow direction relative to the probe, not vessel type. Images were captured as screenshots from the hospital PACS viewer (NilRead; Westlake, OH: Hyland Software) with identifying overlays removed. We cropped and assembled the panels and added arrows and panel letters using Python with the Pillow imaging library (Python Imaging Library); we performed no other image processing. PACS: picture archiving and communication system

On day two, UFH was stopped at 08:22 for mechanical thrombectomy involving the right popliteal, femoral, and iliac veins, lower inferior vena cava (IVC), and left common femoral vein. Procedural heparin was administered. Four enoxaparin administrations were documented under a 1 mg/kg every 12 h order on actual weight across days two to three.

CT obtained on day three to investigate occult malignancy identified a new 1.8 cm left main pulmonary artery embolus, described radiologically as a saddle embolus, additional bilateral emboli, an RV/LV ratio of 1.01, and lower-lobe infarction/atelectasis. The following morning, oxygen saturation was 91% on approximately 36% inspired oxygen, heart rate: 109 beats/min, and respiratory rate: 25 breaths/min, with preserved blood pressure. A retrievable IVC filter was placed on day four for recurrent pulmonary embolism despite anticoagulation; a retrieval date was not documented, and staged iliac venography with intravascular ultrasound was planned at six to 12 weeks. She transferred to a tertiary center on day seven. Representative CT images from day 0 and day three are shown in Figures 2A-2C; the left main pulmonary artery embolus itself is not displayed in the figure.

**Figure 2:**
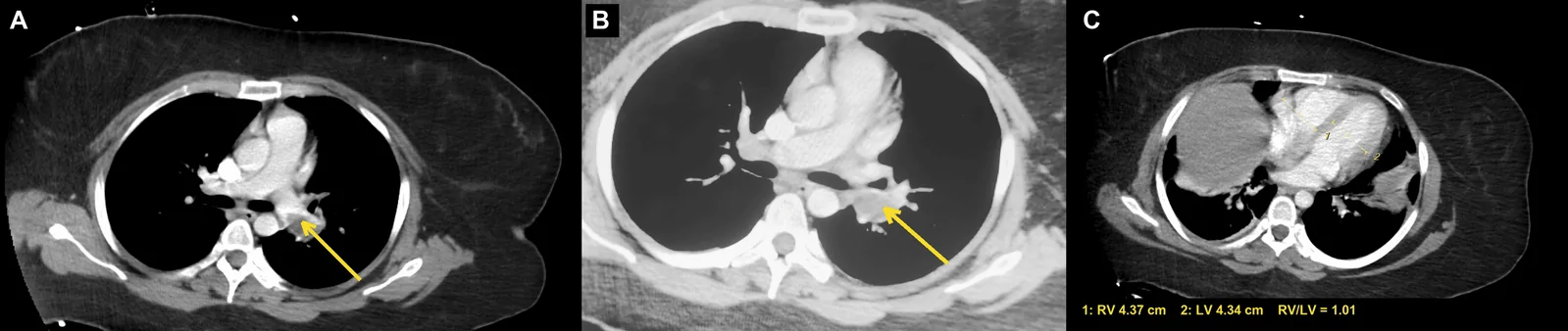
Contrast-enhanced CT of the chest on day 0 and day three. (A) Day 0 (readmission), axial image at the level of the lower lobe pulmonary arteries. A filling defect (yellow arrow) is present in the left lower lobe pulmonary artery, with contrast opacification around it, consistent with non-occlusive pulmonary embolism; the radiology report documented a decreased overall embolic burden compared with the index study. (B) Day three, axial image at a comparable level: a left lower lobe pulmonary arterial filling defect (yellow arrow), consistent with pulmonary embolism, on the study that reported the new embolic event. (C) Day three, axial image at the level of the ventricles, with the interpreting radiologist’s measurements as stored in the PACS viewer: (1) right ventricle, 4.37 cm; (2) left ventricle, 4.34 cm; RV/LV ratio, 1.01. The calipers were not placed by the author; the measurement values are reproduced beneath the image, and their interpretation is addressed in the Discussion section. Panels A and C were captured as screenshots from the hospital PACS viewer (NilRead; Westlake, OH: Hyland Software), with identifying overlays removed; panel B was captured from a recording of the same viewer display and converted to grayscale. We cropped and assembled the panels, added the arrows, panel letters, and text line beneath panel C, and reproduced the stored measurement values using Python and the Pillow imaging library (Python Imaging Library); we performed no other image processing. RV: right ventricle; LV: left ventricle; PACS: picture archiving and communication system

Anticardiolipin and anti-β2-glycoprotein I IgG exceeded the assay reporting limit on days -13 and three; corresponding IgM results were elevated. These samples were 16 days apart, so persistence for at least 12 weeks had not been established for antiphospholipid syndrome (APS) classification [5]. The treating teams nevertheless recorded a clinical diagnosis of APS. LA testing was not performed initially and was uninterpretable on day three because anticoagulant activity was detected. That sample, collected approximately 2 h after enoxaparin, had aPTT: 137.4 s, normal thrombin time: 15.4 s, and detectable anti-Xa activity. ANA was 1:160 speckled; complement was normal, and anti-double-stranded DNA results differed between assays. Factor V Leiden and prothrombin mutations were absent; pregnancy testing was negative. All eight paired aPTT/anti-Xa samples followed detection of the new PE (Table 2). With a UFH anti-Xa target of 0.3-0.7 IU/mL, three values were below range, two at its lower boundary and three above the lower boundary but within range [1]. On day nine, aPTT was 176 s with anti-Xa 0.34 IU/mL and same-day INR 1.1; the tertiary laboratory associated anti-Xa 0.3-0.7 IU/mL with aPTT 60-100 s. That sample was collected at 05:27 on the day the regimen changed from UFH to enoxaparin; the exact administration times at the tertiary center were not supplied, so whether the sample reflected UFH or enoxaparin exposure could not be established.

**Table 2:** Paired activated partial thromboplastin time and heparin anti-Xa results after recurrent pulmonary embolism. Day 0 denotes readmission with phlegmasia cerulea dolens. The new pulmonary embolus was detected on day three; all pairs were collected afterward. UFH anti-Xa target: 0.3-0.7 IU/mL. INR was 2.1 on day five, 1.8 on day six, and 1.1 on day nine. Argatroban was stopped at 09:17 on day four, and UFH was restarted at 09:18. aPTT: activated partial thromboplastin time; UFH: unfractionated heparin; INR: international normalized ratio

Day (time)	Laboratory	aPTT (s)	Anti-Xa (IU/mL)	Anti-Xa interpretation
4 (15:36)	Referring	>200	0.41	Within range
4 (23:50)	Referring	198.9	0.30	Lower boundary
5 (06:42)	Referring	183.4	0.20	Below range
5 (18:08)	Referring	183.4	0.30	Lower boundary
6 (00:37)	Referring	185.8	0.25	Below range
6 (06:28)	Referring	196.6	0.25	Below range
6 (17:59)	Referring	>200	0.66	Within range
9 (05:27)	Tertiary	176	0.34	Within range

INR was 2.7 on day three, then 4.5 during argatroban on day four. On day seven, it was 1.7 at the referring center and 1.2 at the tertiary center approximately 18 h later. These were separate samples. Reagent identities, chromogenic factor X, factor II activity, and alternative-reagent PT results were unavailable.

Platelets were 300×10⁹/L on day seven, 84×10⁹/L on day 10, and 65×10⁹/L on day 11. A heparin-induced thrombocytopenia (HIT) immunoassay sent on day three, when the new embolus was identified during heparin exposure and a switch to argatroban ordered, yielded a negative result. A repeat specimen collected at 18:36 on day 11 was positive for heparin-dependent antiplatelet antibodies with optical density 2.562; the result was reported at 15:12 on day 12. The assay type, manufacturer, and positivity threshold were not stated in the available reports. A serotonin release assay (SRA) was ordered, but its result was unavailable.

Additional investigations showed C3 121 mg/dL and C4 32 mg/dL, within their respective reference intervals (Table 3). Anti-double-stranded DNA was 26 IU/mL at the referring laboratory and 14 IU at the tertiary laboratory; the different methods and thresholds preclude treating these as a single quantitative trend. Anemia studies on day three included iron 25 µg/dL (reference: 50-212 µg/dL), total iron-binding capacity 209 µg/dL (228-428 µg/dL), transferrin saturation 12% (20-55%), ferritin 386 ng/mL (11-307 ng/mL), and vitamin B12: 292 pg/mL. A positive direct antiglobulin test was reported, but haptoglobin was elevated, and a final diagnosis of autoimmune hemolytic anemia was not established in the available records. These results were obtained on different dates, not as one simultaneous panel.

**Table 3:** Laboratory findings during the initial presentation, recurrent thrombosis, and early platelet recovery. Day 0 denotes readmission; day -13 is the initial presentation; day three is the subsequent pulmonary embolism evaluation; and day 13 is early recovery. Values in the one-day column may come from different collection times. Reference intervals are those printed by the indicated laboratory; tertiary intervals must not be used to reclassify results from the referring laboratory. Conventional CBC and chemistry units are harmonized for display. Serial paired aPTT/anti-Xa results appear in Table 2. The repeat HIT specimen collected on day 11 was positive (optical density: 2.562), reported on day 12; the SRA result was unavailable. Platelets on days 10, 11, and 12 were 84×10⁹/L, 65×10⁹/L, and 79×10⁹/L, respectively. Additional ANA titers and patterns were not performed. Duplicate calculated neutrophil outputs in the source extracts are not separate specimens. AI: antibody index; eGFR: estimated glomerular filtration rate; IFA: indirect immunofluorescence assay; DRVVT: dilute Russell viper venom time; SCT: silica clotting time; ANA: antinuclear antibody; MCV: mean corpuscular volume; MCH: mean corpuscular hemoglobin; MCHC: mean corpuscular hemoglobin concentration; RDW: red cell distribution width; AST: aspartate aminotransferase; ALT: alanine aminotransferase; TNP: test not performed; aPTT: activated partial thromboplastin time; PT: prothrombin time; INR: international normalized ratio; HIT: heparin-induced thrombocytopenia; SRA: serotonin release assay; NR: unit or reference interval not reported in the supplied extract; -: no result provided for that date

Test	Units	Reported reference interval	Day -13	Day 3	Day 13
White blood cells	×10⁹/L	3.9-11.0 (tertiary)	11.35	8.97	10.1
Red blood cells	×10¹²/L	3.88-5.37 (tertiary)	4.54	3.21	3.51
Hemoglobin	g/dL	11.7-15.8 (tertiary)	12.92	9.00	10.1
Hematocrit	%	36.0-49.0 (tertiary)	38.5	26.5	31.3
MCV	fL	80.0-99.0 (tertiary)	84.9	82.7	89.2
MCH	pg	27.0-34.0 (tertiary)	28.5	28.1	28.8
MCHC	g/dL	32.0-35.7 (tertiary)	33.5	34.0	32.3
RDW	%	11.6-15.0 (tertiary)	13.9	14.3	16.1
Platelets	×10⁹/L	150-450 (tertiary)	186	253	115
Mean platelet volume	fL	8.9-12.8 (tertiary)	7.9	7.8	10.8
Neutrophils	%	NR	74.1	70.5	55.3
Lymphocytes	%	NR	14.5	17.6	26.3
Monocytes	%	NR	9.6	9.7	9.9
Eosinophils	%	NR	1.2	1.7	3.2
Basophils	%	NR	0.6	0.5	0.5
Absolute neutrophils	×10⁹/L	1.50-7.40 (tertiary)	8.4	6.3	5.61
Absolute lymphocytes	×10⁹/L	1.30-3.90 (tertiary)	1.6	1.6	2.66
Absolute monocytes	×10⁹/L	0.30-1.10 (tertiary)	1.1	0.9	1.00
Absolute eosinophils	×10⁹/L	0.0-0.50 (tertiary)	0.1	0.2	0.32
Absolute basophils	×10⁹/L	0.0-0.20 (tertiary)	0.1	0.0	0.05
Nucleated red blood cells	/100 WBC	0.0-0.5 (tertiary)	-	-	0.0
Immature granulocytes	%	NR	-	-	4.8
Absolute immature granulocytes	×10⁹/L	0.0-0.10 (tertiary)	-	-	0.49
Immature platelet fraction	%	0.9-11.2 (tertiary)	-	-	5.0
Absolute immature platelet fraction	×10⁹/L	NR	-	-	5.80
Sodium	mmol/L	135-145 (tertiary)	133	134	137
Potassium	mmol/L	3.5-5.2 (tertiary)	3.8	3.7	4.0
Chloride	mmol/L	98-108 (tertiary)	101	104	105
Carbon dioxide	mmol/L	24-32 (tertiary)	25.3	25.0	26
Anion gap	mmol/L	3-11 (tertiary)	6.7	5.0	6
Blood urea nitrogen	mg/dL	6-20 (tertiary)	12	9	14
Creatinine	mg/dL	0.40-1.20 (tertiary)	0.7	0.5	0.71
eGFR	mL/min/1.73 m²	≥60 (tertiary)	>60	>60	119
Calcium	mg/dL	8.6-10.6 (tertiary)	9.2	8.8	9.1
Glucose	mg/dL	70-110 (tertiary)	112	99	103
Magnesium	mg/dL	NR	-	1.8	2.1
Phosphorus	mg/dL	NR	-	2.6	3.4
Albumin	g/dL	3.4-5.0 (tertiary)	4.3	-	3.5
Total protein	g/dL	6.0-8.0 (tertiary)	7.7	-	6.4
Alkaline phosphatase	U/L	40-125 (tertiary)	78	-	100
AST	U/L	10-40 (tertiary)	13	-	54
ALT	U/L	7-50 (tertiary)	29	-	100
Total bilirubin	mg/dL	0.0-1.2 (tertiary)	0.7	-	0.4
Direct bilirubin	mg/dL	≤0.2 (tertiary)	-	-	0.2
High-sensitivity troponin I	NR	NR	<2 on two samples	-	-
D-dimer	NR	NR	3,477	-	-
PT, routine assay	s	12.0-15.0 (tertiary)	17.5	30.1	14.2
INR	dimensionless	0.9-1.2 (tertiary)	1.5	2.7	1.1
PT, antiphospholipid panel	s	10.1-13.1 (referral)	20.0	26.6	-
aPTT	s	25.0-36.0 (referral)	95.5; panel TNP	137.4	-
Thrombin time	s	10.3-16.6 (referral)	TNP	15.4	-
Anti-Xa screening assay	Qualitative	Not present	TNP	Present	-
Fibrinogen	mg/dL	163-463 (referral)	TNP	Not indicated	-
DRVVT screen and 1:1 screen mixture	Ratio	0.0-1.20 (referral)	TNP	Not indicated	-
DRVVT confirmation	Total ratio	0.0-1.20 (referral)	TNP	Not indicated	-
DRVVT 1:1 confirmation mixture	Ratio	0.0-1.20 (referral)	TNP	Not indicated	-
SCT screen and 1:1 screen mixture	Ratio	0.0-1.22 (referral)	TNP	Not indicated	-
SCT confirmation	Total ratio	0.0-1.22 (referral)	TNP	Not indicated	-
SCT 1:1 confirmation mixture	Ratio	0.0-1.22 (referral)	TNP	Not indicated	-
Lupus anticoagulant interpretation	Qualitative	Not applicable	TNP	Invalid because of anticoagulant interference	-
Anti-β2-glycoprotein I IgG	U/mL	0.0-19.9	>112.0	>112.0	-
Anti-β2-glycoprotein I IgM	U/mL	0.0-19.9	28.8	40.5	-
Anticardiolipin IgG	GPL U/mL	0.0-19.9	>112.0	>112.0	-
Anticardiolipin IgM	MPL U/mL	0.0-19.9	28.1	37.9	-
Factor V Leiden mutation	Qualitative	Negative	Negative, homozygous normal genotype	-	-
Prothrombin gene mutation	Qualitative	Negative	Negative, homozygous normal genotype	-	-
Protein C antigen	NR	NR	87	-	-
Free protein S antigen	NR	NR	85	-	-
ANA screen by IFA	Qualitative	Negative	-	Positive	-
ANA titer	Titer	<1:80 negative; 1:80 low; >1:80 elevated	-	1:160	-
ANA pattern	Qualitative	Not applicable	-	Nuclear, speckled	-
Anti-double-stranded DNA, referral assay	IU/mL	≤4 negative; 5-9 indeterminate; ≥10 positive	-	26	-
Anti-Scl-70	AI	<1.0 negative	-	<1.0	-
Anti-Smith	AI	<1.0 negative	-	<1.0	-
Anti-Sm/RNP	AI	<1.0 negative	-	<1.0	-
Anti-SSA	AI	<1.0 negative	-	<1.0	-
Anti-SSB	AI	<1.0 negative	-	<1.0	-
HIT immunoassay	Qualitative	Antibody not detected	-	Negative	-
Ferritin	ng/mL	11-307	-	386	-
Serum iron	µg/dL	50-212	-	25	-
Unsaturated iron-binding capacity	µg/dL	155-300	-	184	-
Total iron-binding capacity	µg/dL	228.0-428.0	-	209.0	-
Transferrin saturation	%	20-55	-	12	-
Vitamin B12	pg/mL	NR	-	292	-
Erythrocyte sedimentation rate	NR	NR	-	52	-
Reticulocyte count	NR	NR	-	2.10	-
Haptoglobin	NR	NR	-	328	-
Lactate dehydrogenase	NR	NR	-	212	-
Urine pregnancy test	Qualitative	Negative	Negative	-	-
Urine color/appearance	Qualitative	NR	Yellow/turbid	-	-
Urine specific gravity	Dimensionless	NR	1.035	-	-
Urine pH	Dimensionless	NR	5.5	-	-
Urine glucose/blood/nitrite	Qualitative	NR	All negative	-	-
Urine bilirubin/ketones	Qualitative	NR	Both negative	-	-
Urine urobilinogen	Qualitative	NR	Normal	-	-
Urine protein	Qualitative	NR	Trace	-	-
Urine leukocyte esterase	NR	NR	250	-	-
Urine red blood cells	NR	NR	0-2	-	-
Urine white blood cells	NR	NR	6-10	-	-
Urine epithelial cells/bacteria	Qualitative	NR	Few/few	-	-
Urine mucus	Qualitative	NR	Occasional	-	-

Argatroban ran from 00:54 to 09:17 on day four while HIT was initially investigated. After the negative immunoassay, UFH restarted at 09:18 with anti-Xa-guided titration. Supplementary boluses were recorded on days five to six. Methylprednisolone 40 mg intravenously every 8 h began on day four as empiric treatment for antiphospholipid syndrome with recurrent thrombosis despite anticoagulation; catastrophic APS was not diagnosed, and it was stopped on day eight after tertiary rheumatology review. Repeat echocardiography showed preserved right ventricular function; further thrombectomy was deferred.

On day nine, UFH was changed to enoxaparin 80 mg every 12 h and warfarin 2.5 mg daily. The reduced enoxaparin dose, approximately 0.7 mg/kg, was documented as a response to body mass index above 40. No low-molecular-weight heparin-calibrated peak anti-Xa level was measured on this regimen; a single later anti-Xa result (1.21 IU/mL on day 11) lacked a recorded dosing interval and was not interpretable. On day 11, enoxaparin and warfarin were stopped, and fondaparinux 10 mg subcutaneously daily was started for suspected HIT. Figures 3A, 3B summarize the sequence.

**Figure 3:**
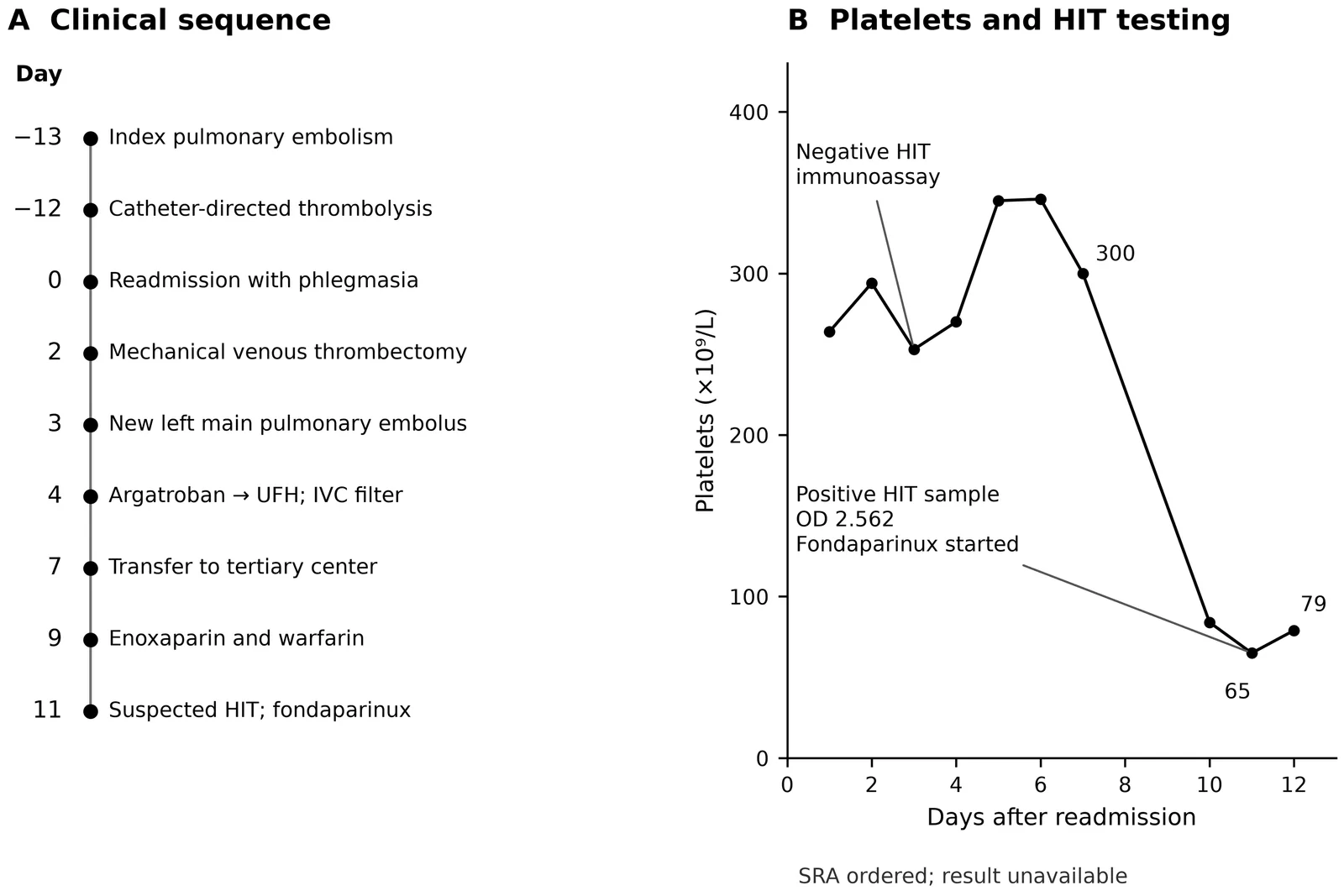
Clinical sequence and platelet course. Day 0 denotes readmission with phlegmasia. (A) Key clinical events in order; vertical spacing is schematic, not proportional to elapsed time. (B) Plots dated platelet counts and marks collection of the negative HIT immunoassay on day three and the later positive sample on day 11. The positive result was reported on day 12. Lines connecting observations guide the eye and do not represent additional measurements. Platelets were 300×10⁹/L on day seven, 65×10⁹/L on day 11, and 79×10⁹/L on day 12. The later positive immunoassay supported the treating team's HIT diagnosis; its optical density was 2.562, and the SRA result was unavailable. The figure was created by the authors of this study from the clinical records using Python with the Matplotlib library; no image from any other source was used. PE: pulmonary embolism; IVC: inferior vena cava; UFH: unfractionated heparin; HIT: heparin-induced thrombocytopenia; SRA: serotonin release assay

Leg symptoms improved, although exertional dyspnea persisted. Platelets increased to 79×10⁹/L on day 12 and 115×10⁹/L on day 13. The discharge plan was fondaparinux 10 mg daily for three months, followed by reassessment for outpatient warfarin with hematology follow-up. The receiving hematology team attributed the earlier recurrence to interrupted bridging and subtherapeutic anticoagulation; referring notes raised concern about INR reliability. Available records did not resolve these interpretations. Long-term outcome, SRA results, and antibody testing at least 12 weeks later were unavailable.

## Discussion

An inhibitor such as LA was a plausible contributor to the disproportionate aPTT prolongation but was not confirmed. Other considerations included anticoagulant effects, factor deficiencies, acute-phase influences, and preanalytical problems. Reduced factor II activity can contribute to aPTT/anti-Xa discordance [6]. Argatroban was confirmed during the INR of 4.5 and confounded its interpretation [7]. Despite the normal thrombin time, recent enoxaparin administration and detectable anti-Xa activity limited interpretation of LA testing [2].

The principal finding is marked aPTT/anti-Xa discordance in a patient whose aPTT was already prolonged before documented heparin. Such discordance is not novel - disproportionately elevated aPTT occurred in 42% of 2321 paired measurements in one hospital series [8]. Here, the day-nine pair remained discordant with INR 1.1, so a vitamin K antagonist effect is an unlikely explanation for that sample; because its heparin exposure (UFH or enoxaparin) could not be established, it does not, by itself, measure UFH intensity. It did not identify a specific inhibitor. Displayed infusion orders cannot establish that aPTT results caused underdosing, and the post-recurrence pairs cannot establish anticoagulant intensity before the PE. Takemoto et al. investigated biochemical contributors to discordance, including reduced factor II activity [6]. This patient’s records demonstrate discordance but do not include the factor studies needed to establish its biochemical explanation.

These observations are consistent with APS monitoring guidance favoring anti-Xa when baseline aPTT is prolonged [1,2]. However, anti-Xa must be interpreted using the administered anticoagulant, sampling time, and assay calibration. A UFH target cannot be applied indiscriminately to enoxaparin or fondaparinux, and anti-Xa does not quantify warfarin or argatroban effect [1,2]. Here, every paired measurement followed detection of recurrent PE. Because no low-molecular-weight heparin-calibrated peak level was measured, we cannot establish whether the reduced day-nine enoxaparin regimen achieved therapeutic exposure in class III obesity; reduced weight-based dosing may be subtherapeutic in recurrent venous thromboembolism (VTE). The findings therefore support a monitoring concern, not proof that inadequate UFH exposure preceded the embolus.

Warfarin monitoring warrants independent assessment. Most appropriately calibrated, LA-insensitive thromboplastins support reliable INR monitoring in APS; an unreliable aPTT does not automatically invalidate INR [9]. The elevated early PT and concern about recurrence during apparently therapeutic warfarin justified further assessment. Comparing suitable thromboplastins and chromogenic factor X can characterize selected discrepancies, as demonstrated in a recent case report [10]. However, neither measurements from separate samples 18 h apart nor subtraction of a baseline INR proves interference. Warfarin dose alone does not establish inadequate treatment. No independent result demonstrated INR overestimation in this patient.

For recurrence, inadequate anticoagulant exposure during outpatient bridging, genuine breakthrough thrombosis, and embolization around thrombectomy remained possibilities. Periprocedural embolization has been reported during iliocaval thrombectomy, but detection after the procedure did not establish its timing or mechanism here [11]. Procedural heparin was documented, and the absence of an inferior vena cava (IVC) filter at thrombectomy did not establish whether other embolic protection was used. The receiving hematology service attributed recurrence to interrupted bridging and subtherapeutic anticoagulation. The unavailable original outpatient and admission INR records prevent independent adjudication. The later reduced enoxaparin regimen began on day nine, six days after the new PE was detected, and cannot explain that earlier event.

For the later platelet decline, HIT, consumption, and autoimmune thrombocytopenia were considered. Hematology assigned a 4Ts score of 5 (reconstructed by the authors from the recorded data as thrombocytopenia 2, timing 2, thrombosis 0, and other causes 1) and diagnosed HIT from the clinical course and strongly positive immunoassay [12]. Platelets rose to 345-346×10⁹/L on days five to six during corticosteroid treatment and fell after corticosteroid withdrawal on day eight, so corticosteroid withdrawal cannot be separated from HIT as a contributor. No new thrombus extension was documented during the platelet fall.

A negative HIT immunoassay describes the tested specimen; it does not permanently exclude HIT during continued heparin exposure. New thrombocytopenia warrants reassessment of clinical probability and repeat testing when indicated [3]. The change from a negative day-three result to a positive day-11 result does not establish when pathogenic antibodies developed. Higher optical density increases the likelihood of platelet-activating antibodies, although interpretation depends on the assay [12]. Pauzner et al. documented antigen-positive, functionally negative results in patients with APS or systemic lupus erythematosus [4]. Thus, the optical density of 2.562 and clinical course support the treating team’s concern, while the unavailable serotonin release assay (SRA) limits functional confirmation. Neither result establishes that HIT caused the earlier PE.

Adediran and Agostino described a woman with recurrent extensive DVT and PE, antiphospholipid antibodies, and HIT who received argatroban followed by fondaparinux and later warfarin [13]. Their report included a positive SRA and persistent antiphospholipid antibodies at three months [13]. The present case shares the difficulty of recurrent thrombosis during successive anticoagulant regimens and subsequent use of fondaparinux but lacks those confirmatory follow-up results. This comparison highlights why clinical HIT, functional HIT confirmation, and persistent APS antibodies should be reported separately.

Alternative anticoagulation must address both acute HIT and longer-term thrombotic risk. American Society of Hematology (ASH) guidance supports stopping heparin and initiating a non-heparin anticoagulant; selection depends on clinical stability, bleeding risk, organ function, and procedural needs. Fondaparinux is an option in clinically stable patients, whereas short-acting parenteral agents may be preferable when urgent procedures are anticipated. Vitamin K antagonists should not be initiated during acute HIT before platelet recovery, usually to at least 150×10⁹/L [3]. The documented day-11 cessation of enoxaparin and warfarin and initiation of fondaparinux are consistent with these principles.

A change to argatroban does not automatically resolve aPTT interference. In spiked plasma samples, Love et al. found that aPTT overestimated direct thrombin inhibitor concentrations in the presence of lupus inhibitors, whereas a plasma-diluted thrombin time performed more reliably [14]. This laboratory study supports discussion with the coagulation laboratory about a locally validated drug-specific alternative when baseline aPTT is unsuitable. Such testing was not documented during this patient’s brief argatroban exposure. Her INR of 4.5 during that infusion cannot be used to establish warfarin intensity [7].

Kang et al. retrospectively compared fondaparinux with argatroban or danaparoid in suspected or confirmed HIT and found no statistically significant differences in thrombosis or bleeding after propensity matching [15]. The observational design and mixed population limit direct extrapolation to concurrent APS and HIT. The patient’s fondaparinux regimen therefore represents an individualized clinical strategy, not evidence of superiority. Long-term treatment also requires separate reassessment - European Alliance of Associations for Rheumatology (EULAR) recommends vitamin K antagonists for thrombotic APS and cautions against rivaroxaban in triple-positive APS [16]. LA was not confirmed here, so this patient should not be described as triple-positive.

The course also illustrates practical reasons for multidisciplinary coordination. Hematology reassessed the platelet decline and anticoagulant strategy; rheumatology evaluated the autoimmune findings and discontinued corticosteroids; and cardiology assessed whether further intervention was warranted. These are documented contributions, not evidence that coordination itself improved outcomes. A useful approach in similar cases is a shared timeline of administered drugs, procedures, imaging, and assay results, with laboratory and pharmacy input when monitoring or dosing is uncertain. At discharge, one explicit plan should identify the anticoagulant, monitoring method, follow-up clinician, and responsibility for retrieving pending HIT and APS studies. This approach is consistent with guideline emphasis on treatment adherence, INR monitoring, and patient education [16].

Limitations remain substantial and include incomplete outpatient anticoagulant records, unavailable original admission INR, uninterpretable LA testing, no independent assessment of INR reliability, and unavailable SRA and long-term antibody results. The available hemolysis, autoimmune, and inherited thrombophilia investigations are summarized in Table 3, with unreported assay details identified. These results do not exclude every competing diagnosis. The day-three CT right ventricular-to-left ventricular diameter ratio of 1.01 met the threshold of 1.0 or greater associated with right ventricular dysfunction on CT pulmonary angiography, whereas repeat echocardiography showed preserved right ventricular function; the available records do not reconcile the two assessments [17]. The retrievable filter placed on day four addressed recurrence during anticoagulation, a setting in which filters have limited supporting evidence, and the documented follow-up addressed iliac venous imaging rather than a retrieval date [17]. The strongest conclusion is a documented monitoring discrepancy followed by a new clinical HIT assessment, rather than proof of a single mechanism connecting every thrombotic event.

## Conclusions

This case demonstrates marked aPTT/anti-Xa discordance in a patient with antiphospholipid antibodies and a subsequent clinical diagnosis of HIT. It supports early selection of an appropriate UFH monitoring assay, independent assessment of INR reliability, and renewed HIT evaluation when the platelet trajectory changes. Interpretation of recurrence requires the actual anticoagulant exposure and procedural timeline. The records do not establish that assay interference caused recurrent thrombosis, and functional HIT confirmation and long-term APS testing remain unavailable.
